# Identification of benign and malignant breast nodules on ultrasound: comparison of multiple deep learning models and model interpretation

**DOI:** 10.3389/fonc.2025.1517278

**Published:** 2025-02-18

**Authors:** Xi Wen, Hao Tu, Bingyang Zhao, Wenbo Zhou, Zhuo Yang, Lijuan Li

**Affiliations:** ^1^ Department of Ultrasound, The Central Hospital of Enshi Tujia And Miao Autonomous Prefecture (Enshi Clinical College of Wuhan University), Enshi, China; ^2^ Department of Neurology, China-Japan Union Hospital of Jilin University, Changchun, China; ^3^ Department of Stomatology, China-Japan Union Hospital of Jilin University, Changchun, China

**Keywords:** weakly supervised deep learning, diagnosis, breast tumor, ultrasonography, interpretability

## Abstract

**Background and Purpose:**

Deep learning (DL) algorithms generally require full supervision of annotating the region of interest (ROI), a process that is both labor-intensive and susceptible to bias. We aimed to develop a weakly supervised algorithm to differentiate between benign and malignant breast tumors in ultrasound images without image annotation.

**Methods:**

We developed and validated the models using two publicly available datasets: breast ultrasound image (BUSI) and GDPH&SYSUCC breast ultrasound datasets. After removing the poor quality images, a total of 3049 images were included, divided into two classes: benign (N = 1320 images) and malignant (N = 1729 images). Weakly-supervised DL algorithms were implemented with four networks (DenseNet121, ResNet50, EffientNetb0, and Vision Transformer) and trained using 2136 unannotated breast ultrasound images. 609 and 304 images were used for validation and test sets, respectively. Diagnostic performances were calculated as the area under the receiver operating characteristic curve (AUC). Using the class activation map to interpret the prediction results of weakly supervised DL algorithms.

**Results:**

The DenseNet121 model, utilizing complete image inputs without ROI annotations, demonstrated superior diagnostic performance in distinguishing between benign and malignant breast nodules when compared to ResNet50, EfficientNetb0, and Vision Transformer models. DenseNet121 achieved the highest AUC, with values of 0.94 on the validation set and 0.93 on the test set, significantly surpassing the performance of the other models across both datasets (all P < 0.05).

**Conclusion:**

The weakly supervised DenseNet121 model developed in this study demonstrated feasibility for ultrasound diagnosis of breast tumor and showed good capabilities in differential diagnosis. This model may help radiologists, especially novice doctors, to improve the accuracy of breast tumor diagnosis using ultrasound.

## Introduction

Ultrasound (US) is widely used in the clinical diagnosis of breast lesions, particularly for differentiating between benign and malignant breast nodules (1, 2). However, breast ultrasound diagnosis is inherently limited by inter-observer variability, especially among non-experts (3–6). This variability often leads to higher false-positive or false-negative rates, resulting in unnecessary biopsies and surgeries or a delay in treatment (5). To mitigate these issues, deep learning (DL) techniques have been progressively introduced into breast ultrasound diagnosis (7).

Traditional deep learning approaches generally rely on a fully supervised learning paradigm, requiring image annotation, which typically involves manual delineation of regions of interest (ROIs) corresponding to the lesions (8, 9). Even with the implementation of automatic ROI segmentation methods, manual verification of the segmentation results is still necessary. Given that deep learning is a data-driven technique, the image annotation process is labor-intensive and time-consuming, the potential for subjective bias introduced by human judgment, which may affect the model’s performance (9).

Recent studies have explored deep learning methods that eliminate the need for ROI annotation by directly inputting the entire image for training and diagnostic prediction (10–14). The previous study has shown that the weakly supervised DL algorithm provided excellent diagnostic performance that was not inferior to the fully supervised DL algorithm with manual and automated annotation (12). In this approach, the model learns and classifies based on the global features of the full ultrasound image, thereby eliminating the dependency on manual annotation. This may streamline data preparation and diminish subjective biases, potentially enhancing model training efficiency and clinical generalizability.

However, studies based on deep learning with weak supervision in breast ultrasound imaging remains limited. Therefore, this study aims to compare several deep learning models that directly input unsegmented whole breast ultrasound images to differentiate between benign and malignant nodules, and to analyze the interpretability of the prediction results.

## Materials and methods

### Dataset

The breast ultrasound image data used in our study comes from two publicly available datasets: breast ultrasound image (BUSI) [Available online: https://scholar.cu.edu.eg/?q=afahmy/pages/datasetm (access on 1 July 2022)] and GDPH&SYSUCC breast ultrasound [Available online: https://github.com/yuhaomo/HoVerTrans (access on 11 January 2023)] datasets.

**Figure 1:**
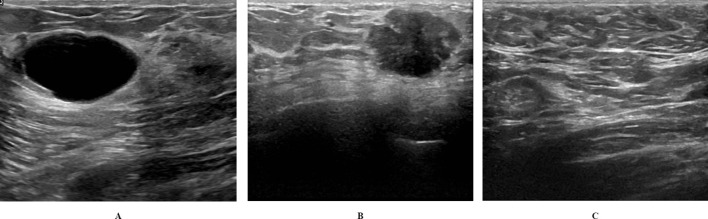
Representative images from BUSI dataset: **(A)** Benign; **(B)** Malignant; **(C)** Normal.

The BUSI dataset is categorized into three classes: benign (N = 487 images), malignant (N = 210 images), and normal (N = 133 images). BUSI dataset images were taken from women between the ages of 25 and 75 years; hence, the dataset is preferred for studies involving early breast cancer detection in women below 40 years of age (15). The dataset was collected in 2018 from 600 female patients. The dataset consists of 780 images, each with an average size of 500 × 500 pixels. The dataset images are PNG files. Representative images from the dataset are shown in **Figure 1**.

**Figure 2:**
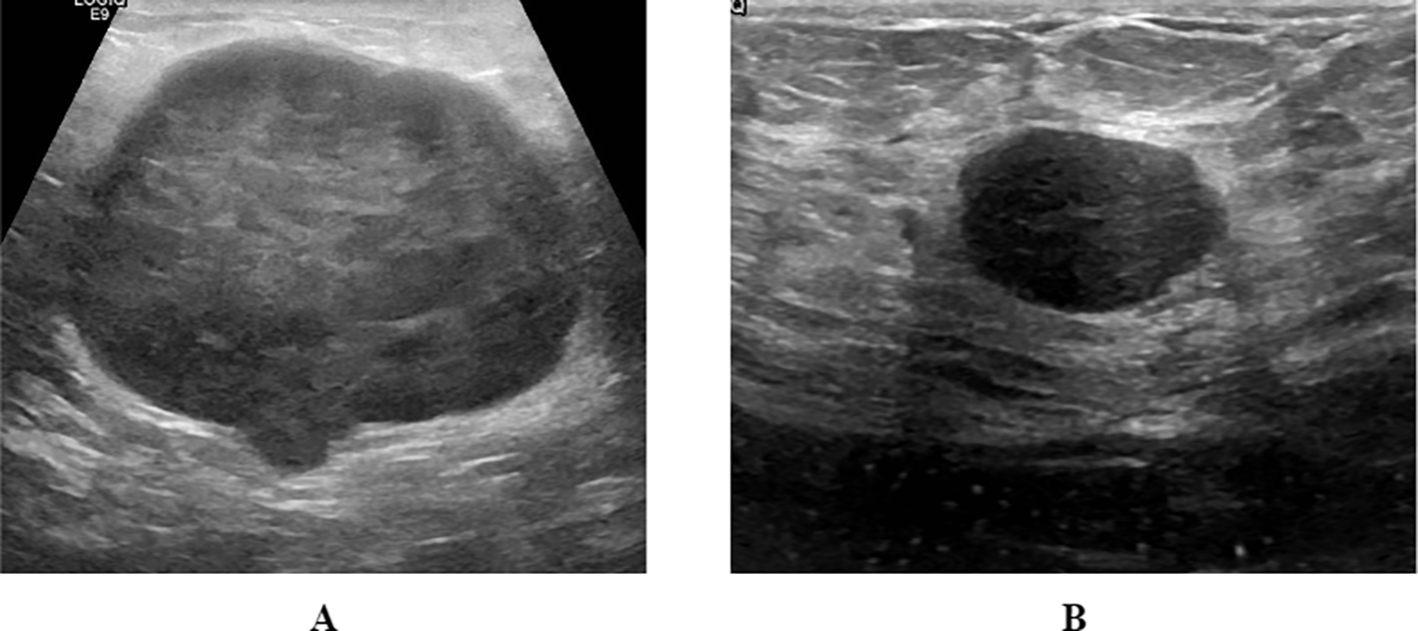
Representative images from GDPH&SYSUCC dataset: **(A)** Malignant; **(B)** Benign.

The GDPH&SYSUCC breast ultrasound dataset is divided into two categories: benign (N = 886 images) and malignant (N = 1519 images) (14). Images were collected from two medical centers: 1) Department of Ultrasound, Guangdong Provincial People’s Hospital (GDPH, Guangzhou, Guangdong, China) and 2) Department of Ultrasound, Sun Yat-sen University Cancer Prevention and Control Centre (SYSUCC, Guangzhou, Guangdong, China). Images and their corresponding BI-RADS scores were exported from a picture archiving and communication system (PACS). Acquisition devices included Hitachi Ascendus (Japan), Myriad DC-80 (China), Toshiba Aplio 500 (Japan), and Sonic Aixplorer (France). All images were classified as benign or malignant based on biopsy or post-operative pathology reports. The average image size was 844 × 627 pixels, ranging from 278 × 215 to 1,280 × 800 pixels. The images in the dataset are in PNG format and of different sizes. Representative images from this dataset are shown in **Figure 2**.

**Figure 3:**
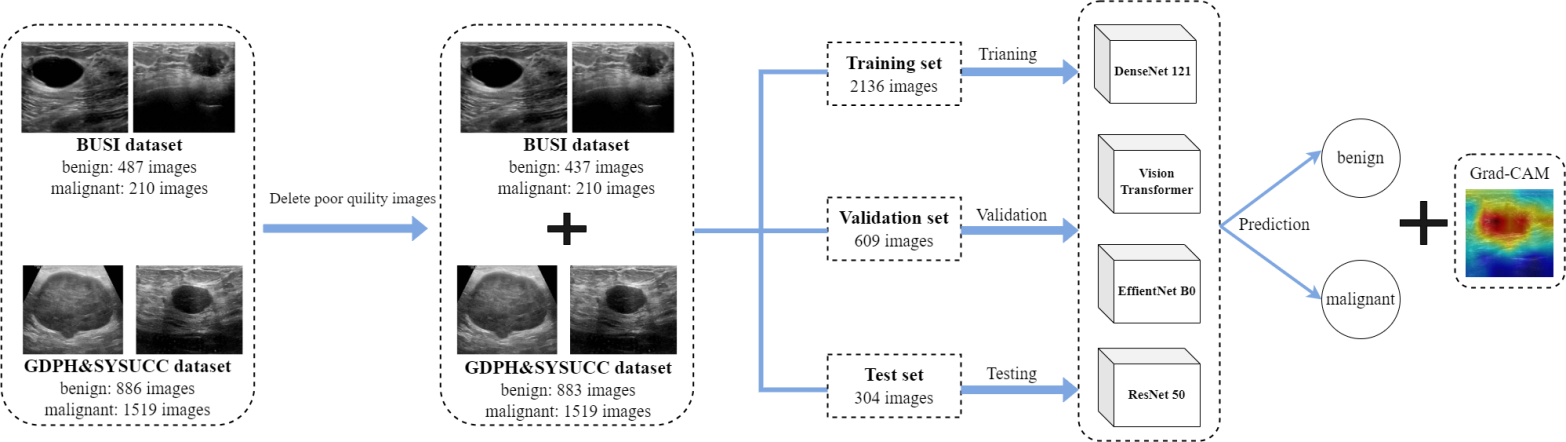
Research flowchart of this study.

The BUSI and GDPH&SYSUCC datasets were combined to form a mixed dataset. Benign images from both datasets were aggregated to establish a mixed benign image class, and a similar procedure was applied to malignant images. Following the exclusion of low-quality images, a total of 3049 images were retained for the models construction and validation. The mixed dataset was divided into benign (N = 1320 images) and malignant (N = 1729 images) classes, then split into training, validation, and test sets in a 7:2:1 ratio (**Figure 3**).

### Preprocessing

The preprocessing of the data involved resizing and normalizing the dataset images. The images in both datasets utilized in this study had different pixel sizes from their corresponding sources. Therefore, we resized them to use them as input in our proposed method. Each image was resized to 224 × 224 pixels. Their pixel values were normalized to the range of [0, 1] by dividing the maximum intensity value. Besides, we performed spatial transformation-based augmentations (horizontal flip, color jitter, sharpening, salt-and pepper, gamma correction, random rotation, and height shift) to equalize the size of the images in each class.

### Deep classification models

For deep classifiers, we employed Vision Transformer (ViT) and three representative convolutional neural networks (CNN) that have achieved state-of-the-art performance in various computer vision tasks: ResNet50, EfficientNetb0, and DenseNet121 (**Figure 3**). Weights were initialized randomly, and all models were trained from scratch, without pretraining. The entire process, from data preprocessing to model building and interpretation, was implemented in the Medical Open Network for Artificial Intelligence (MONAI), a Pytorch-based open-source framework for deep learning in healthcare imaging (16). We selected DenseNet121, ResNet50, EfficientNetB0, and ViT to represent both traditional CNNs and emerging transformer-based models, aiming to explore their performance in breast ultrasound image classification. DenseNet121: Chosen for its efficient feature reuse through dense connections, making it well-suited for extracting subtle features in small medical datasets. ResNet50: Selected for its residual learning capability, which addresses the degradation problem in deep networks and enables robust feature extraction. EfficientNetB0: Included for its computational efficiency and balanced optimization of network depth, width, and resolution, which are beneficial for resource-constrained environments. Vision Transformer: Added to explore the potential of transformer-based models in capturing global contextual information, offering a contrast to CNN architectures. This selection ensures a comprehensive comparison of diverse architectures to identify the most effective approach for breast ultrasound image classification.

### Implementation details

We trained our models for 50 epochs using the Adam optimizer with a learning rate of 0.00001. A batch size of 16 and exponential decay were used for training in the ViT model. GELU was utilized as an activation function with an L2 regularizer for the ViT model. In CNNs, ReLu was utilized with an L2 regularizer. For experiments involving comparison, the same parameter settings were utilized to avoid bias in the results. Each model was modified so that its last classifier layer would be a sigmoid layer to be able to perform two-label classification.

### Reader study

A reader study was conducted on the test set, wherein three radiologists independently assessed the malignant and benign breast lesions using breast ultrasound images. The participating radiologists, designated as Reader 1 (XW), Reader 2 (ZY), and Reader 3 (HT), possessed 2, 5, and 13 years of experience in breast ultrasound, respectively. All readers were blinded to any clinicopathologic information, including model performance and the pathological results associated with the images.

### Evaluation metrics

Metrics including accuracy, area under the receiver operating curve (AUC), F1-score, recall, and precision were used to assess the performance of our model. For differential diagnosis, we used area under the receiver operating characteristics curve (AUC) as the primary metric for comparing the algorithm performance, and the DeLong test of significance for comparing the AUC of two correlated receiver operating characteristics curves (ROCs).

### Model interpretation

According to the evaluation metrics, the optimal model would be selected for model interpretation. To allow an easier interpretation of image feature extraction, we derived activation maps using the gradient-weighted class activation map (Grad-CAM) technique (17).

## Results

### Model performance

**Figure 4:**
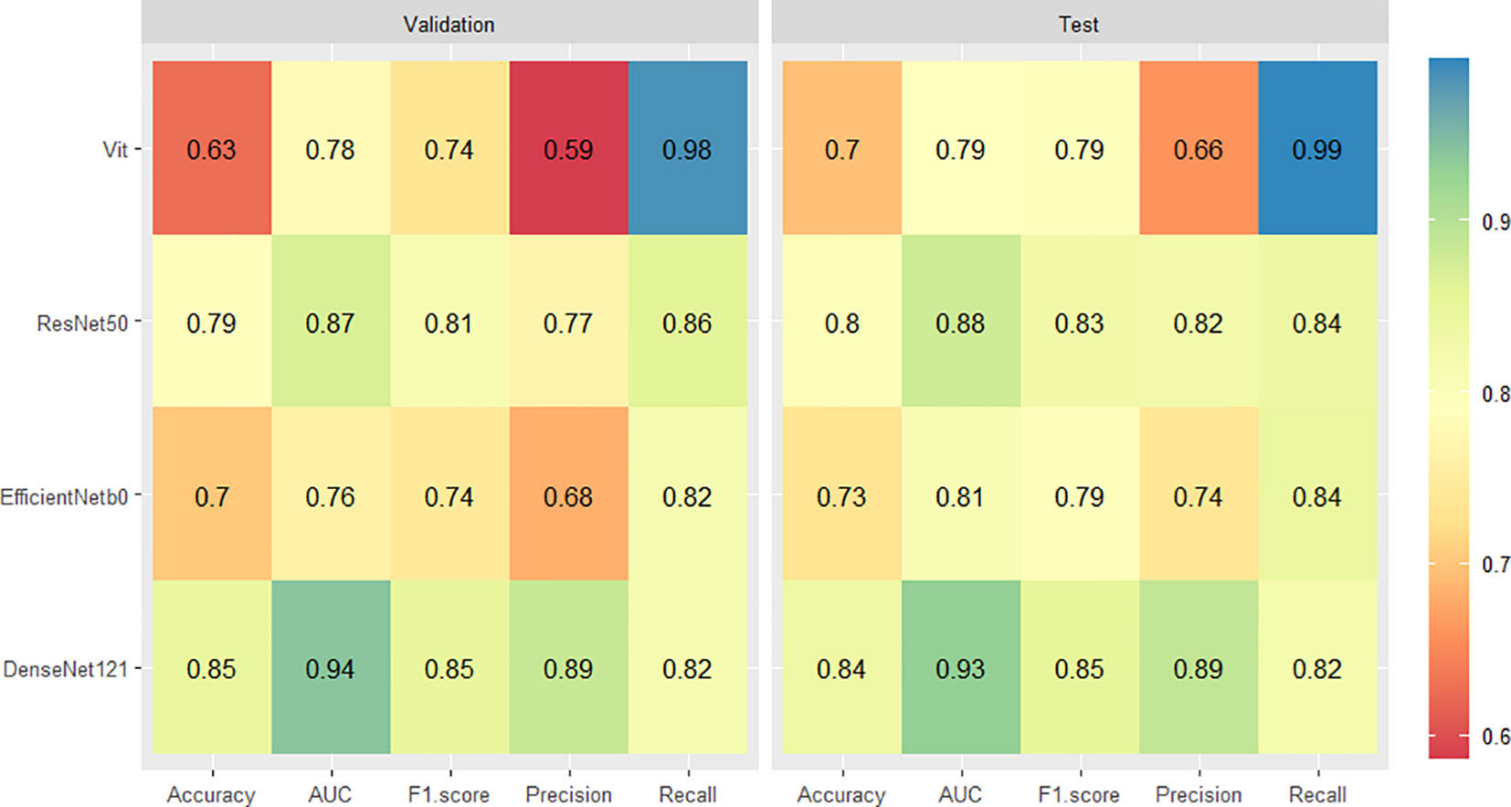
Heatmaps of the diagnostic performance metrics between different DL models in both validation and test sets.

**Figure 5:**
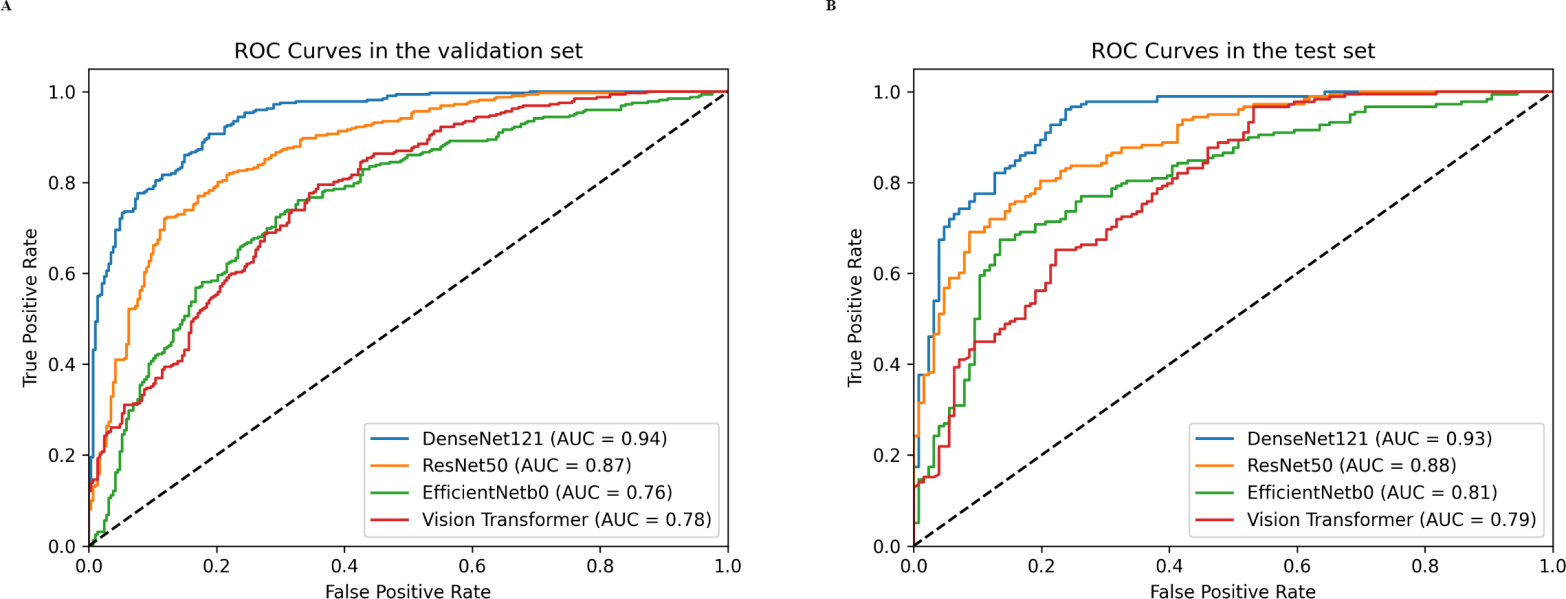
Receiver operating characteristics (ROC) curves of different DL models in both validation **(A)** and test **(B)** sets.

For the validation set, the DenseNet121 model (AUC 0.94, 95% CI 0.92 - 0.96) achieved higher performances in the differential diagnosis between benign and malignant breast masses than ResNet50, EfficientNetb0, and ViT (ResNet50: AUC 0.87, 95% CI 0.84 - 0.90; EfficientNetb0: AUC 0.76, 95% CI 0.72 - 0.80; ViT: AUC 0.78, 95% CI 0.74 - 0.81) (**Figures 4**, **5**). Delong’s test was performed to compare the AUC, showing that the difference between DenseNet121 and other models is significant (all P <0.001). The accuracy, F1 score, precision, and recall of DenseNet121 in the validation set was 0.85, 0.85, 0.89, and 0.82, respectively (**Figure 4**). The accuracy, F1 score, precision, and recall of ResNet50 in the validation set was 0.79, 0.81, 0.77, and 0.86, respectively (**Figure 4**). The accuracy, F1 score, precision, and recall of EfficientNetb0 in the validation set was 0.70, 0.74, 0.68, and 0.82, respectively (**Figure 4**). The accuracy, F1 score, precision, and recall of ViT in the validation set was 0.63, 0.74, 0.59, and 0.98, respectively (**Figure 4**).

For the test set, the DenseNet121 model (AUC 0.93, 95% CI 0.90 - 0.96) achieved higher performances in the differential diagnosis between benign and malignant breast masses than ResNet50, EfficientNetb0, and ViT (ResNet50: AUC 0.88, 95% CI 0.84 - 0.92; EfficientNetb0: AUC 0.81, 95% CI 0.75 - 0.85; ViT: AUC 0.79, 95% CI 0.74 - 0.84) (**Figure 4**, **5**). Delong’s test was performed to compare the AUC, showing that the difference between DenseNet121 and other models is significant (all P <0.05). The accuracy, F1 score, precision, and recall of DenseNet121 in the test set was 0.84, 0.85, 0.89, and 0.82, respectively (**Figure 4**). The accuracy, F1 score, precision, and recall of ResNet50 in the test set was 0.80, 0.83, 0.82, and 0.84, respectively (**Figure 4**). The accuracy, F1 score, precision, and recall of EfficientNetb0 in the test set was 0.73, 0.79, 0.74, and 0.84, respectively (**Figure 4**). The accuracy, F1 score, precision, and recall of ViT in the test set was 0.70, 0.79, 0.66, and 0.99, respectively (**Figure 4**).

### Performance of different readers

**Figure 6:**
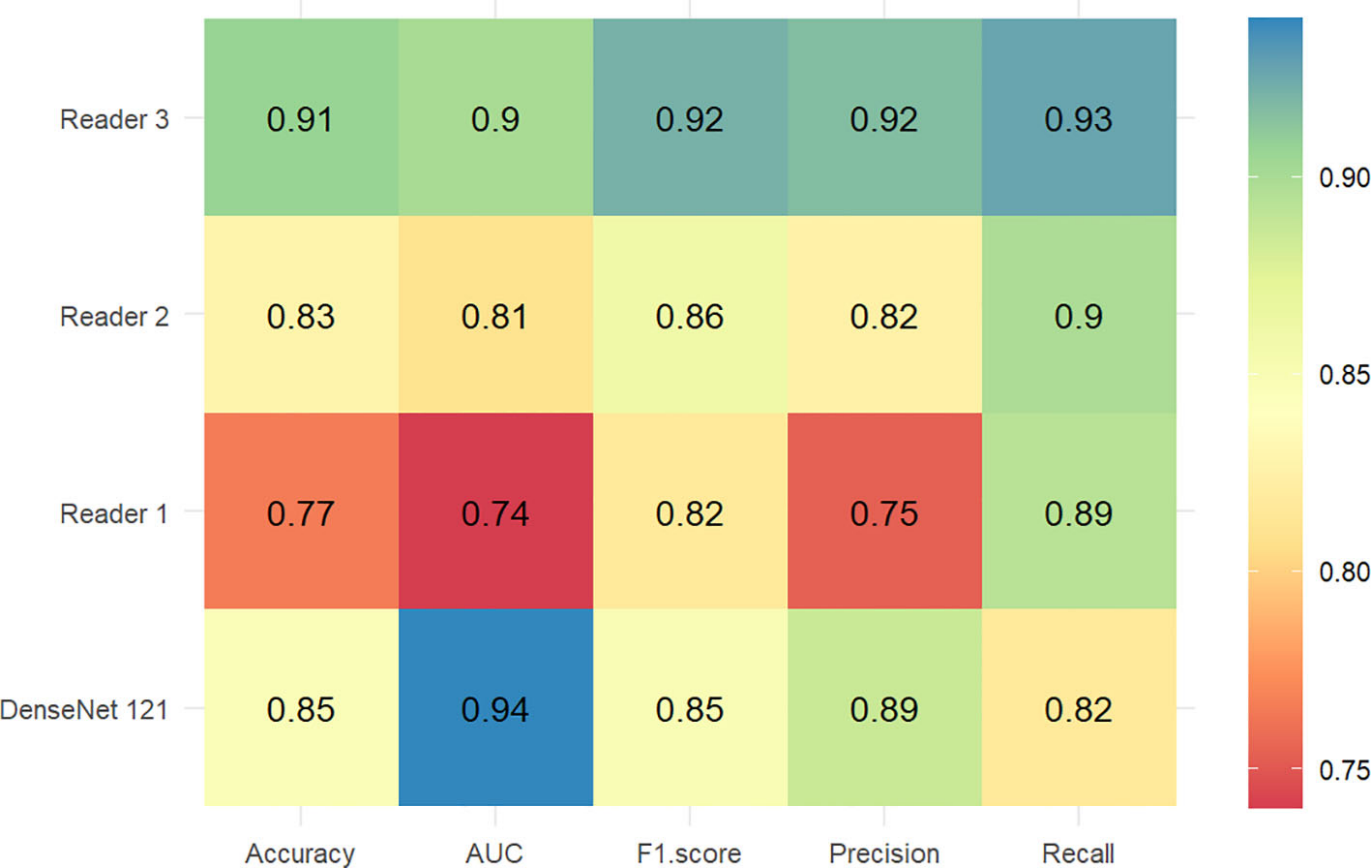
Heatmaps of the diagnostic performance metrics between the DenseNet121 model and ultrasound physicians with different experience levels in the test set.

**Figure 6** compares the diagnostic accuracy of the DenseNet121 models with ultrasound physicians of varying experience in predicting breast nodule benignity or malignancy. The performance of the DenseNet121 model (AUC 0.93, 95% CI 0.90 - 0.96) in predicting breast lesions benignity or malignancy outperformed the reader 1 (AUC 0.74, 95% CI 0.69 - 0.79) and reader 2 (AUC 0.82, 95% CI 0.77 - 0.86). The reader 3 (AUC 0.90, 95% CI 0.87 - 0.94) and the DenseNet121 model showed similar performance.

## Discussion

In this study, we demonstrated that the DenseNet121 model, using full-image input without ROI annotations, achieved superior diagnostic performance in differentiating between benign and malignant breast nodules compared to ResNet50, EfficientNetb0, and ViT. Specifically, the DenseNet121 model showed the highest AUC (0.94 in the validation set and 0.93 in the test set), significantly outperforming the other models in both validation and test datasets (all P < 0.05). The DenseNet121 model demonstrated comparable performance with experienced readers in breast ultrasound diagnosis. These findings suggest that DenseNet121 model utilizing full-image input are effective for breast ultrasound diagnosis, may reducing the need for time-consuming manual ROI annotation while maintaining high diagnostic accuracy.

Ultrasound (US) is the preferred diagnostic modality for breast lesions owing to its simplicity and non-invasive nature. Nonetheless, the assessment of breast lesions using US is limited by interobserver variability and frequently depends on the subjective experience of the radiologist (3–6). Due to the excellent performance of deep learning on images, the integration of deep learning with medical imaging is receiving increasing attention from medical practitioners (7). Most DL models currently in clinical practice are trained in a supervised manner, requiring humans to annotate images by drawing the ROI lesion (8, 9). Even with automated ROI segmentation, manual verification remains essential. The removal of ROI annotation could be advantageous, as conventional methods that depend on manual or automated segmentation necessitate substantial human involvement and may introduce variability into the training data. Furthermore, the characterization of the tumor lesion’s edges is critical for accurately distinguishing between benign and malignant lesions. The segmentation of ROI may lead to the omission of this essential edge information. Consequently, further study is necessary to explore the role of weakly supervised deep learning algorithms that process the entire image without ROI segmentation in differentiating between benign and malignant ultrasound breast lesions.

Previous studies have demonstrated that the weakly-supervised DL algorithms were feasible for US diagnosis of breast cancer with well-performing localization and differential diagnosis (12, 18, 19). Consistent with these findings, our study showed that the weakly-supervised DL algorithm, which circumvents the need for ROI annotation by utilizing the entire ultrasound image as input, is effective in distinguishing between benign and malignant breast lesions. Thus, this method is promising as an efficient and cost-effective tool for assisting radiologists, especially novice radiologists, in breast tumor diagnosis. In large-scale breast cancer screening programs, the weakly-supervised DL algorithms alleviate the workload by automating the initial diagnostic process for extensive image datasets, thereby assisting radiologists in the rapid exclusion of non-suspicious lesions (20). Furthermore, the weakly-supervised DL algorithms may be integrated into computer-aided diagnosis (CAD) systems to analyze patients’ ultrasound images in real time, offering preliminary assessments of benign or malignant characteristics concurrent with the radiologist’s examination. Radiologists can utilize the model’s predictions as a reference point, synthesizing them with their own clinical expertise to formulate a comprehensive evaluation, thereby enhancing the accuracy and efficiency of diagnostic processes. In addition, deep learning-based interpretative techniques, including Grad-CAM and Occlusion Sensitivity Analysis, can emphasize regions of an image that possess significant diagnostic value, thereby prompting radiologists to focus more intently on lesion characteristics that are pertinent to the diagnosis of breast tumor.

Prior studies have demonstrated the efficacy of the DenseNet121 model in differentiating between benign and malignant breast lesions (10, 19). In our study, the DenseNet121 model also showed higher performance for differentiating between benign and malignant breast lesions using ultrasound imaging in both validation and test sets than other classical deep learning models (e.g., ResNet50 and EfficientNetb0), indicating its robustness and generalizability. Moreover, DenseNet121 model’s precision (0.89) and recall (0.82) in the validation set, as well as similar values in the test set, reflect its ability to accurately classify malignant lesions while minimizing false positives and negatives, thereby reducing the rate of unnecessary biopsies. This consistent performance across different datasets highlights the DenseNet121 model’s potential for real-world clinical implementation, where variability in ultrasound equipment and imaging conditions is common. Some studies have shown that Transformer-based models can outperform CNNs in diagnostic tasks, particularly when trained on large-scale datasets or using pre-trained weights on ImageNet (21–24). The Vision Transformer architecture benefits from its ability to capture global contextual features through attention mechanisms, making it advantageous in tasks involving complex patterns or large datasets. Nevertheless, in our study, the Vision Transformer underperformed compared to DenseNet121, factors (1): The ResNet50, and EfficientNetB0. This result may be attributed to several Vision Transformer model is highly dependent on extensive datasets to acquire effective global representations, while our training dataset comprised only 2136 images. Conversely, CNNs demonstrate greater robustness with smaller datasets due to their efficient extraction of local features. (2) In contrast to CNNs, which exhibit relatively low sensitivity to initialization, the Vision Transformer architecture encompasses a substantial number of parameters and generally gains from pre-training on extensive datasets. Consequently, training the Vision Transformer from scratch on our limited dataset likely hindered its performance. (3) Breast ultrasound imaging predominantly depends on texture and edge information for the prediction of malignancy. CNNs are more adept at capturing these local features. In contrast, the global attention mechanism of Vision Transformers may not fully leverage these patterns in this context. Future work may explore transfer learning with pre-trained Transformer models, data augmentation, or hybrid architectures like Swin Transformer to better leverage the strengths of Transformers in breast ultrasound diagnosis.

**Figure 7:**
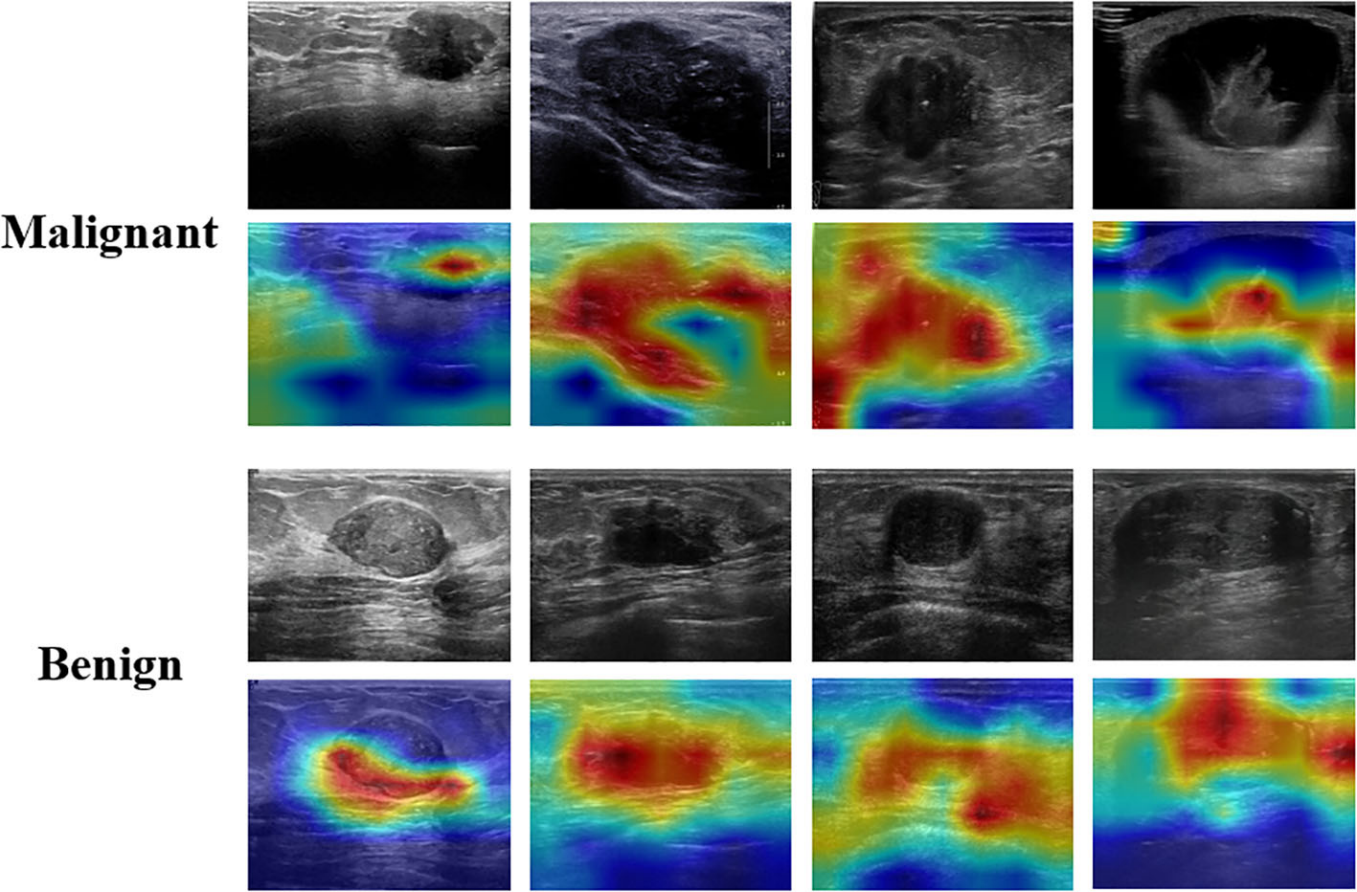
Interpretation of the weakly supervised DenseNet121 model using the gradient-weighted class activation map (Grad-CAM).

Given that the majority of deep learning neural networks function as “black boxes”, this study employs Grad-CAM to enhance the interpretability of the DenseNet121 model (**Figure 7**). Grad-CAM serves not only as a critical interpretative tool for the automated classification of ultrasound breast lesions but also contributes to improve the clinical applicability of the model. Grad-CAM shows the regions within ultrasound images that most significantly influence the classification outcome, thereby enhancing the transparency of the neural networks’ complex decision-making processes for radiologists. This increased transparency aids radiologists in comprehending the model’s rationale, thereby mitigating blind reliance on machine learning algorithms and fostering greater acceptance among radiologists. In instances where breast lesions present with indistinct boundaries or subtle characteristics, radiologists may encounter challenges in establishing definitive diagnoses. Grad-CAM generates heatmaps that emphasize regions of diagnostic importance, thereby facilitating the rapid identification of features deemed critical by the model. This information can be integrated with the radiologist’s expertise, fostering a more comprehensive and reasonable decision-making process. Grad-CAM serves as a valuable tool for radiologists in evaluating potential inaccuracies in the model’s predictions. When the highlighted areas do not align with established characteristics of breast lesions, radiologists are prompted to critically assess the reliability of the model’s output. This feedback mechanism aids in mitigating misjudgments arising from image noise or extraneous features, thereby reducing the risk of diagnostic errors.

### Limitations

Our study has many limitations. (1) While the DenseNet121 model demonstrated good performance on the validation and test sets across diverse imaging conditions, its clinical reliability and generalizability are constrained by the limited dataset size and the absence of independent external validation. Future research should employ larger, multi-institutional datasets from various ultrasound devices to more effectively validate the robustness and generalizability of the DenseNet121 model. (2) Although our study highlights the efficiency gained by eliminating ROI annotations, a direct comparison of the time and labor savings achieved by full-image input versus region-based methods was not conducted. Future studies should aim to quantify these practical advantages. (3) Treatment options vary according to the type of pathology in benign and malignant breast lesions. The lack of specific pathological types prevents further prediction of the pathological types of benign and malignant breast lesions. Future studies should focus on this aspect. (4) Due to the retrospective nature of our study, the model was built and evaluated based on retrospective data, and its clinical reliability still needs to be validated with prospective data.

## Conclusion

This study developed and validated a deep learning algorithm that uses full-image input without ROI annotation to differentiate between benign and malignant breast nodules in breast ultrasound images. The weakly supervised DenseNet121 model exhibited superior diagnostic performance compared to other models. Thus, this method is promising as an efficient and less labor-intensive tool for assisting radiologists, especially novice radiologists, in breast tumor diagnosis.

## Data Availability

The datasets presented in this study can be found in online repositories. The names of the repository/repositories and accession number(s) can be found below: Breast ultrasound image (BUSI) [Available online: https://scholar.cu.edu.eg/?q=afahmy/pages/datasetm (access on 1 July 2022)] and GDPH&SYSUCC breast ultrasound [Available online: https://github.com/yuhaomo/HoVerTrans (access on 11 January 2023)] datasets.
